# Evolution and genomic organization of the insect sHSP gene cluster and coordinate regulation in phenotypic plasticity

**DOI:** 10.1186/s12862-021-01885-8

**Published:** 2021-08-04

**Authors:** Megan Leask, Mackenzie Lovegrove, Abigail Walker, Elizabeth Duncan, Peter Dearden

**Affiliations:** 1 Department of Biochemistry, University of Otago, Dunedin, New Zealand; 2 Genomics Aotearoa and Department of Biochemistry, University of Otago, Dunedin, New Zealand; 3 School of Biology, Faculty of Biological Sciences, University of Leeds, Leeds, LS2 9JT UK

**Keywords:** Phylogenetics, sHSP, Honeybee, Birth-and-death evolution, Synteny, Arthropods, H3K27me3

## Abstract

**Background:**

Conserved syntenic gene complexes are rare in Arthropods and likely only retained due to functional constraint. Numerous sHSPs have been identified in the genomes of insects, some of which are located clustered in close proximity. Previous phylogenetic analyses of these clustered sHSP have been limited to a small number of holometabolous insect species and have not determined the pattern of evolution of the clustered sHSP genes (sHSP-C) in insect or Arthropod lineages.

**Results:**

Using eight genomes from representative insect orders and three non-insect arthropod genomes we have identified that a syntenic cluster of sHSPs (sHSP-C) is a hallmark of most Arthropod genomes. Using 11 genomes from Hymenopteran species our phylogenetic analyses have refined the evolution of the sHSP-C in Hymenoptera and found that the sHSP-C is order-specific with evidence of birth-and-death evolution in the hymenopteran lineage. Finally we have shown that the honeybee sHSP-C is co-ordinately expressed and is marked by genomic features, including H3K27me3 histone marks consistent with coordinate regulation, during honeybee ovary activation.

**Conclusions:**

The syntenic sHSP-C is present in most insect genomes, and its conserved coordinate expression and regulation implies that it is an integral genomic component of environmental response in arthropods.

**Supplementary Information:**

The online version contains supplementary material available at 10.1186/s12862-021-01885-8.

## Background

Evolutionary conserved syntenic gene complexes are relatively rare in arthropod genomes, with only three major well-characterised examples, the Hox complex [1–6], the E(spl)-C [7, 8] and the runt complex [9] represented in the majority of arthropod lineages. Those complexes, in which gene-order remains conserved over evolutionary time, are likely to be constrained by factors such as coordinated regulation (as in the case of the Hox and E(Spl)-C). Most gene complexes (though not all) [10], are formed by duplication of a single ancestor, but the evolutionary history of such complexes is often complicated with instances of duplication, loss, and gene conversion. In this study we examine the evolutionary history and gene expression patterns of a syntenic cluster of small heatshock protein encoding genes (sHSP-C) [11] in the genomes of arthropods.

sHSPs are found throughout the three domains of life and these proteins act as molecular chaperones that bind misfolded proteins. The sHSPs are one of the most up-regulated classes of heat-shock proteins following stress [12], have critical roles in normal development in *Drosophila* [13]*.* The sHSPs are categorized as having molecular weights between 12 and 42 kDa and are comprised of a conserved α-crystallin domain of about 100 amino acid residues and a highly variable N-terminal domain [14, 15]. The sequence diversity of the N-terminal region has been proposed to be responsible for regulation of expression, species-specific functions, cellular localisation or tissue specific expression [11, 16, 17] and oligomerization and chaperoning capacity [18].

The most well studied insect sHSP are seven sHSP genes contained in a syntenic cluster at a 15 kb region at the 67B locus in *D. melanogaster* and the sHSP *lethal (2) essential for life (l(2)efl)* found non-linked on Chromosome 2R. The *Drosophila* sHSP-cluster (sHSP-C) genes and non-linked *l(2)efl* all have similar sequences in their promoter regions, with multiple heat shock elements (HSE) and binding sites for Broad-Complex (BR-C), an ecdysone regulated transcription factor [19, 20]. This implies that the sHSP-C genes and *l(2)efl* share a common ancestral ortholog as well as similar functions and expression [21–23] and therefore the genes of the sHSP-C could be retained in this cluster due to functional constraint. Supporting this conjecture, the seven *D. melanogaster* sHSPs in the sHSP-C have coordinated expression in response to temperature shock, while having distinct expression patterns during normal development [24–27].

sHSP-C genes have also been identified and studied in other insects [11] and coordinated expression of the entire sHSP-C is evident in *Bombyx mori* (Lepidoptera) [11] and *Bemisia tabaci* (Hemiptera) [28]. Genomic organisation of the insect sHSP-C and coordinated expression of sHSP-C genes under certain conditions implies that the sHSP-C is a conserved entity in insects that could be functionally constrained. However, phylogenetic analyses to date which are restricted to the holometabola imply that these sHSP clusters are species-specific and that the sHSPs have undergone multiple independent expansion events in the evolution of insects [11, 28]. Only very recently do phylogenetic analyses in Lepidoptera indicate that the sHSP-C is stable between species of this order [29, 30]. In the absence of more comprehensive expression data and phylogenetic evidence spanning the entirety of the insect orders including the more basally branching holometabola and hemimetabola it is unclear how the sHSP-C has evolved throughout the insect lineage and/or whether coordinate expression suggestive of functional constraint is a feature of the sHSP-C in all insects.

To disentangle the evolutionary relationships of the sHSP-C in insects and assess if the insect sHSP-C evolution could be due to constraint we combine a broad phylogenetic study with a focussed study of the expression of these genes in the basally branching holometabola honeybees. Using a comprehensive suite of arthropod genomes our analyses show that the sHSP-C is an ancestral component of pan-crustacean genomes and its complex evolution throughout arthropod evolution is due to both evolutionary constraint of an ancestral cluster as well as multiple expansion events in different species. Using two tractable models of phenotypic plasticity in the Honeybee we assess whether the sHSP-C is a genomic regulatory domain that is expressed co-ordinately in response to environmental stimuli to further support the notion that the insect sHSP-C is a conserved and functionally constrained environmentally responsive cluster of genes.

## Results

### Phylogenetics

#### A sHSP-cluster is a hallmark of arthropod genomes

In this study, we examine several classes of small heat-shock proteins (sHSPs) which all share an extensive amount of genetic and protein sequence similarity. Herein our analyses contain (1) sHSP genes found syntenically at the same genomic location in a sHSP-cluster (sHSP-C) (2) non-linked sHSPs which are not syntenically clustered with other sHSP genes and (3) *l(2)efl* genes which are non-linked sHSP previously identified to be conserved throughout insects. Previous phylogenetic analyses have identified clustered sHSP genes in the holometabolous insects *Apis mellifera*, *Tribolium castaneum, Bombyx mori*, and *Drosophila melanogaster* [11]. Here, we extended these analyses with the addition of representative species from the hemimetabolous insects *Ladona fulva, Ephemera danica*, *Zootermopsis nevadensis*, *Pediculus humanus*, and include non-insect arthropods *Tetrachynus urticae* [chelicerate]; *Daphnia pule*x [crustacea]; *Strigamia maritima* [myriapoda] and the Lophotrochozoan *Biomphalaria glabrata* [mollusca] as a non-arthropod outgroup in order to systematically examine the evolution of these genes and gene cluster in Arthropods. In our analyses all arthropods have multiple sHSP sequences. In the majority of arthropods sHSP genes are found adjacent to each other in gene clusters on contigs or genome scaffolds (Additional file 2: Figure S1). In comparison sHSP were not found to be clustered in a molluscan genome. Thus a sHSP-C appears to be a feature of arthropod genomes (with the exception of the hemimetabolous insect *Zootermopsis*).

#### *l(2)efl* is an insect innovation

**Fig. 1:**
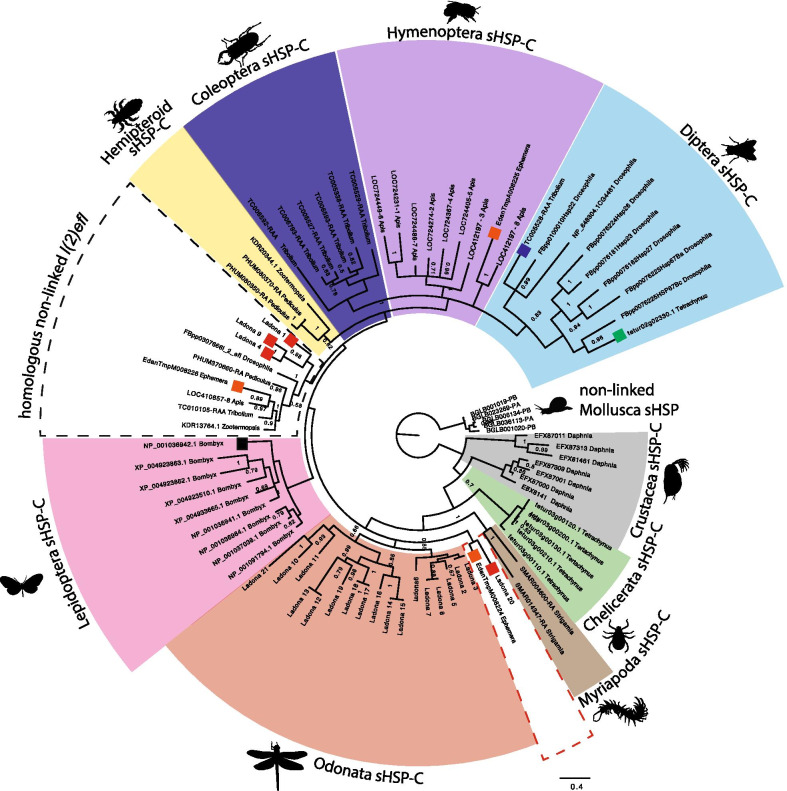
Phylogenetic relationships sHSP genes in Arthropods. Bayesian Phylogram of sHSPs proteins generated using WAG model of amino-acid evolution. Node labels indicate posterior probabilities. The tree is rooted with non-linked sHSP sequences from mollusca. Protein identifiers refer to names in Additional file 1: Table S1. Coloured boxes demarcate the sHSP-C genes from the arthropod orders; diptera (light blue) hymenoptera (purple), coleoptera (dark blue), lepidoptera (pink), hemipteroid lineage (yellow), odonata (red), myriapoda (brown), chelicerata (green) and crustacea (grey). The dashed boxes indicate the non-linked *l(2)efl* sequences in insects

We used BLAST to identify two categories of non-linked sHSPs; those that were most similar to *Drosophila l(2)efl* and non-linked sHSP most similar to the sHSP-C genes. We carried out phylogenetic analyses of the non-linked sHSP genes most similar to *Drosophila l(2)efl* and the genes of the sHSP-C from a single species from each order of the insects, and one species each from chelicerates, crustacean, myriapoda and mollusca (Fig. 1). Since we were focussed on the mechanisms underlying the evolution of the sHSP-C genes and due to the large number of non-linked sHSP that are most similar to the sHSP-C genes these non-linked sHSP genes were excluded from further analyses of the Arthropod sHSPs.

Phylogenetic analysis examining the relationships of the sHSP-C genes and *l(2)efl* indicates the existence of a clade containing the orthologs of the *D. melanogaster l(2)efl* gene from each insect species (Fig. 1, indicated by black dashed box). Examining the genomic organisation of sHSP-C genes we find that the *l(2)efl* gene is not clustered with other sHSP in the holometabolous and hemipteroid insects. However, the ortholog of *l(2)efl* of the basally branching Palaeoptera *Ladona* and *Ephemera* is syntenically clustered with the sHSP-C genes (Additional file 2: Figure S1). In *Ladona* there are three copies of *l(2)efl* clustered in two separate sHSP-C and in *Ephemera* one of the sHSP-C genes falls into the orthologous insect *l(2)efl* phylogenetic group. We find no evidence for orthologs of *l(2)efl* in the genomes of non-insect arthropods thus the *l(2)efl* gene appears to be an innovation specific to insects.

#### The insect sHSP-C shares a common ancestor with *l(2)efl*

Consistent with previous data [11], the majority of the arthropod sHSP-C genes fall into order-specific phylogenetic clades (coloured boxes on Fig. 1) implying that these sHSP-C genes may be the result of group-specific expansion events from an ancestral gene or genes. There are, however, two exceptions. One *Tribolium* sHSP-C gene (dark blue square) and one *Tetrachynus* non-linked sHSP paralog (green square) form a clade with the sHSP-C genes from *Drosophila* (light blue box). Finally with the exception of the *Bombyx* and Odonata sHSP-C genes (which form two sister clades to all other holometabolous and hemipteran insect sHSPs), all of the insect sHSP-C genes share a common branch-point with the homologous non-linked *l(2)efl* genes (dashed box Fig. 1). The phylogenetic signal suggests that the ancestral insect *l(2)efl* was contained in a sHSP-C as evidenced by *l(2)efl* found clustered in Odanata and Ephemera*.* To further support this notion we do observe that *l(2)efl* is found on the same chromosome as the sHSP-C in Hymenoptera, however is separated by 8 Mb (Additional file 2: Figure S1). It is possible that the insect sHSP-C arose from a duplication event that happened when *l(2)efl* was contained in an ancestral sHSP-C. Also noteworthy is that our analyses indicate that the evolution of the sHSP-C in Lepidoptera was different from that of the other holometabolous insects and that this is inconsistent with the current understanding of species relationships. This inconsistency in the phylogenetic signal could be explained by a differential duplication of an ancestral sHSP-C, could be the result of tree reconstruction error or could be the result of gene recombination/conversion between *l(2)efl* and the sHSP-C genes before separation of the ancestral complex or after separation exclusive to the Lepidoptera lineage.

#### sHSP-C copy number is conserved in holometabolous insects

The phylogenetic evidence shows that there is significant flux in the numbers of sHSP-C genes in the insect genomes. Copy number appears to be retained in the holometabolous insects (6–7 sHSP-C genes) whereas in the hemimetabolous insects it is unclear whether the *Ladona* expansion is a lineage specific event, or if the sHSP-C has been reduced (or lost in the case of *Zootermopsis*) in hemipteran genomes. The apparent clade-specific expansions of the sHSP-C genes and the complexity of the phylogeny make it difficult to identify the ancestral sHSP-C gene(s) and thus trace the evolution of the sHSP-C in the insect lineage. Consistent with previous analyses carried out by Li et al. the relationships seen in the phylogram are best explained by independent expansion events of the sHSP-C genes in each of the insect orders. However the species investigated in these initial analyses here and by Li et al. diverged 300 my ago thus the phylogenetic distance between species may be too large to provide sufficient resolution to understand the true relationships of the sHSP-C between species. To combat this issue we investigated the genome organization of the sHSP-C and carried out phylogenetic analyses of the sHSP-C genes in the more closely related species in the hemipteran (hemimetabolous sister group to the holometabola) and hymenopteran (most basally branching holometabolous insects) to deduce the origins of the insect sHSP-C.

#### All Hemipteran insects have a reduced or absent sHSP-C

**Fig. 2:**
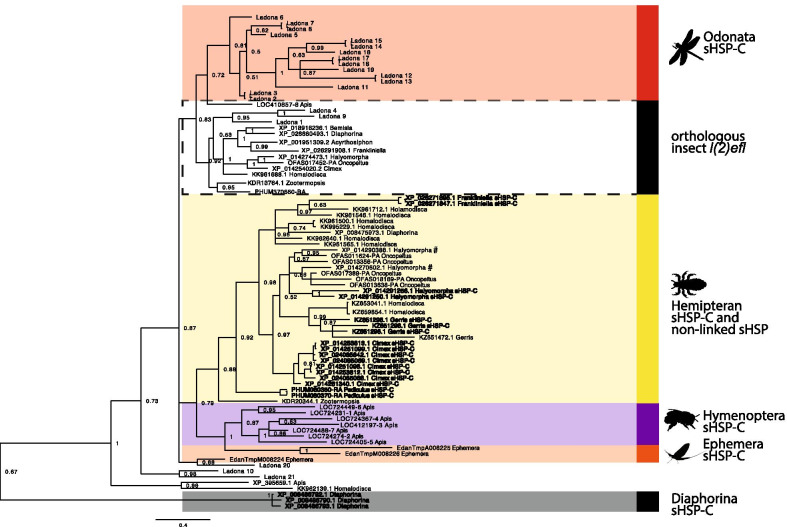
Phylogenetic relationships sHSP genes in Hemipteroid insects. Bayesian Phylogram of sHSPs proteins generated using WAG model of amino-acid evolution. Node labels indicate posterior probabilities. The tree is rooted with the clustered putative alpha-crystallin protein sequences from *Diaphorina citri*. Protein identifiers refer to names in Additional file 1: Table S1. Coloured boxes demarcate the sHSP-C genes from the hemipteroid order (yellow) and the hymenoptera (purple), odanata (red) and ephemera (orange) orders. The dashed boxes indicate the non-linked *l(2)efl* sequences. The dark grey box indicates the sHSP-C sequences from Diaphorina which falls independent of the other hemipteroid sHSP-C sequences in the phylogeny. In the hemipteroid (yellow) sHSP-C sequences are bolded

To assess the evolution of the sHSP-C genes in the hemipteroid lineage we identified sHSP-C genes, *l(2)efl* genes and sHSP paralogs (non-linked sHSP which have more similarity to sHSP-C genes) in hemiptera (Additional file 2: Figure S2) and constructed a phylogram of these plus the honeybee (basally branching holometabolous insect), *Odonata* and *Ephemera* (representative non-hemipteran hemimetabolous insects) sHSP-C genes and *l(2)efl* genes (Fig. 2). With the exception of *Diaphorina citri*, the hemipteroid sHSP-C genes and hemipteroid non-linked *l(2)efl* genes separate into two distinct phylogenetic clades (Fig. 2, yellow box and dashed box, respectively) consistent with our previous arthropod phylogeny (Fig. 1). The hemipteroid sHSP-C genes and non-linked sHSP paralogs share a common branch point with the *Apis mellifera* sHSP-C genes and two of the *Ephemera* sHSP-C genes. Thus the hemiptera sHSP-C is most closely related to the genes of the sHSP-C of *Apis mellifera* (most basally branching holometabolous insect) and *Ephemera* (hemimetabolous insect). In the absence of additional genomes from species that are evolutionary intermediates between holometabola and hemiptera we cannot determine if the hemiptera sHSP-C has been reduced or if sHSP-C gene expansion has occurred in the holometabolous insects. Our conclusion from the phylogeny is that the sHSP-C genes from *Ladona,* excluding those that cluster with *l(2)efl,* appear to be a consequence of lineage specific expansion from non-linked *l(2)efl*.

In hemiptera we see evidence of species-specific duplications of the sHSP-C genes. Within the phylogenetic group of the hemipteroid sHSP-C and non-linked sHSP paralogs each hemipteroid sHSP-C gene is most closely related to a sHSP-C gene from the same species-specific cluster (in bold on Fig. 2). In contrast, the non-linked sHSP paralogs are, in some cases, more closely related to the non-linked sHSP paralogs from closely related species e.g. the non-linked sHSP from *Halyomorpha* and *Oncopeltus* (marked by # on Fig. 2), a closely related Pentatomomorpha species (82 mya diverged [31]).

We found that the *Diaphorina* sHSP cluster, consisting of two genes (Additional file 2: Figure S2), instead aligns with the alpha crystallin genes (excluded from the phylogeny). Additionally the cluster of three sHSP genes from *Diaphorina* group together in a separate clade adjacent to all other insect sHSPs. These data indicate that the clustered genes in *Diaphorina* are instead more similar to the conserved alpha-crystallin genes. The absence of a true sHSP-C in *Diaphorina* is consistent with the absence of a sHSP-C in the other true bug species *A. pisum*.

#### Birth and death evolution of the Hymenoptera sHSP-C

**Fig. 3:**
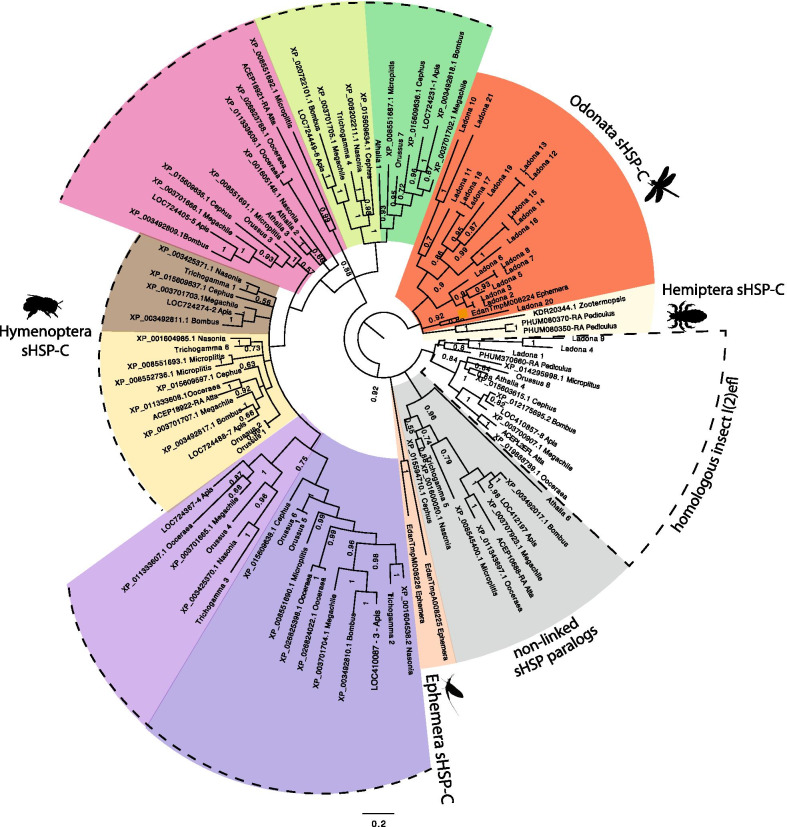
Phylogenetic relationships sHSP genes in Hymenoptera. Bayesian Phylogram of sHSPs proteins generated using WAG model of amino-acid evolution. Node labels indicate posterior probabilities. The tree is rooted with sHSP-C sequences from Palaeoptera (Ephemera and Ladona) insects. Protein identifiers refer to names in Additional file 1: Table S1

sHSP-C genes, non-linked sHSP and *l(2)efl* genes were identified in hymenopteran species (Additional file 2: Figure S3). Comparable to the arthropod phylogeny (Fig. 1) the homologous *l(2)efl* forms a phylogenetic group with all Hymenoptera and other *l(2)efl* genes in other insects (Fig. 3). Additionally, our order specific analyses resolve the phylogenetic signal for the hymenopteran sHSP-C genes. The genomic organization of hymenopteran sHSPs indicates that the sHSP-C is more stable in the hymenopteran lineage than in hemiptera. This is supported by the conservation of orientation of transcription and retention of several genes in the sHSP-C (Additional file 2: Figure S3). Each gene of the Hymenopteran sHSP-C forms a phylogenetic group with representative genes from the other Hymenopteran species, indicating that the expansion of sHSP genes giving rise to the sHSP-C in Hymenoptera are order, rather than species-specific (Fig. 3). The genes of the Hymenoptera sHSP-C likely originate from two ancestral sHSP genes which have duplicated to give rise to a core hymenopteran sHSP-C (dotted box on Additional file 2: Figure S3). This core was present deep in hymenopteran evolution as evidenced by its presence in *Cephus, Orussus* and the Apidae clades which last shared a common ancestor ~ 190 million years ago [32].

In Hymenoptera we find evidence to support birth and death evolution whereby new genes arise from duplication and are either maintained in the genome, deleted or become nonfunctional via deleterious mutations [33]. We see evidence of gene loss in the basal branching sawflies and the ants (Additional file 2: Figure S3) consistent with previous studies [28]. The absence of some of the sHSP-C genes in *Athalia* appears to be due to loss events because we see the full complement of ~ 7 hymenopteran sHSP-C genes in another sawfly *Cephus cinctus*. We also identify remnants of sHSP ORFs in the ant *Atta cephalotes* sHSP cluster (whether these are expressed or functional remains to be determined) whereas the ant *Ooceraea biroi* has retained four of the sHSP-C genes and has another sHSP-C of two genes at another location in the genome. The phylogeny indicates that the genes of the second *Ooceraea* sHSP-C are most similar to that of the first *Ooceraea* sHSP-C. Other examples where genes of the same species sHSP-C are more similar to one another than other Hymenoptera sHSP-C genes are observed twice in *Orussus*, and once in *Athalia* and *Microplitus*, suggestive of recent species-specific duplications.

The cluster observed in *Cephus cinctus* is conserved through to the Apoidea (~ 190 million years diverged) which indicates that the ~ 7 sHSP-C in the extant species of hymenoptera is likely very similar to the ancestral hymenoptera sHSP-C. This analysis indicates that the Hymenopteran sHSP-C was formed early in hymenopteran evolution but has been remodelled, through the birth and death of sHSP-C genes into the clusters seen in different species (Additional file 2: Figure S3).

### Gene expression analyses

#### The sHSP-C is a coordinately regulated gene cluster in *A. mellifera*

Our phylogenetic analyses indicate that in hemimetabolous insects the sHSP complex is relatively unstable. Conversely, our analyses in Hymenoptera indicate that the sHSP complex is conserved over the 190 million years of evolution. Retention of this cluster in Hymenoptera implies that its genomic organisation is constrained and functionally important in some way. Evidence for functional constraint of the sHSP-C has been previously indicated in the higher branching holometabolous insects. In *Drosophila* and Lepidoptera the genes of the sHSP-C are regulated co-ordinately in response to heat-shock and stress [34–36] and in gonadal tissues respectively, analogous to what is seen for the conserved insect E(spl)-C [7] and Hox clusters [37] which are also under functional constraint. Recent phylogenetic evidence in Lepidoptera also indicates that the sHSP-C is conserved in this insect lineage [29, 30]. Thus we hypothesise that coordinated regulation could also explain the maintenance of sHSP clusters in the most basally branching holometabola, hymenoptera. In particular the hymenopteran honeybee displays remarkable and predictable examples of phenotypic plasticity, which we have previously shown can result in a massive transcriptional remodelling and coordinate expression of syntenic gene clusters [38]. To test the possibility that coordinate expression of the sHSP-C genes also occurs in hymenoptera and could be important in phenotypic plasticity, we examined our two previously published RNA-seq datasets [38, 39]. These datasets investigate the differences in gene expression as a result of phenotypic plasticity; the differential feeding of honeybee larvae that results in queen development [39] and the effect of queen mandibular pheromone (QMP) on honeybee ovary activity [38].

In honeybees, the sHSP-C genes are co-ordinately expressed in a data set of gene expression during activation of the honeybee worker ovary (Additional file 2: Figure S4). This coordinate regulation is not seen in datasets of honeybee female larval development, indicating that, as in *Drosophila* and *Bombyx*, these clusters may be regulated in a coordinate way in some situations, and separately in others. This is consistent with the findings of Duncan et al. [38] where they identified a significant number of co-regulated gene clusters associated with the response of adult honeybees to QMP. However, a systematic comparison with four datasets revealed only a very small number of these clusters were co-ordinately regulated in response to different environmental stimuli.

**Fig. 4:**
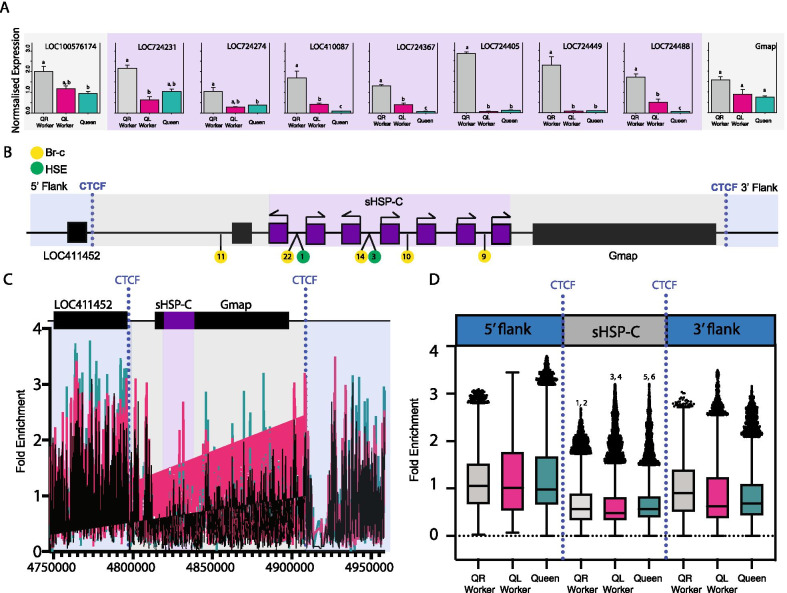
CTCF defines H3K27me3 enrichment at the boundaries of the sHSP-C. **A** The genomic structure at the sHSP-C in honeybees. Regulatory motifs CTCF (blue dotted line), Br-C (yellow circles) and HSE (green circles). **B** Average H3K27me3 enrichment across the sHSP-C. Cyan is queen H3K27me3 enrichment, black is from queen-less workers, and magenta is from queen-right workers. The sHSP-C bound by CTCF (including Gmap and LOC100576174) is significantly depleted of H3K27me3 in queen-right worker, queen-less worker and queen ovaries (Kruskall Wallis with a Dunn post-hoc test) in comparison to the 5ʹ and 3ʹ flanks. Numbers denote statistical significance (p < 0.0001). 1, 3 and 5 are comparisons between the 5ʹ flank and CTCF bound cluster for queen-right, queen-less worker and queens respectively. 2, 4 and 6 are comparisons between the 3ʹ flank and CTCF bound cluster for queen-right worker, queen-less worker and queen respectively. **C** Boxplot illustrating H3K27me3 enrichment across the sHSP-C and the 3ʹ and 5ʹ flanks that are demarcated by CTCF. Boxplot whiskers indicate minimum and maximum, the box is defined by 25th percentile, median, and 75th percentile. Outliers, data points outside 1.5 times the interquartile range above the upper quartile and below the lower quartile, are shown as individual data points. **D** RT-qPCR of genes honeybee sHSP-C as well as immediately adjacent genes Gmap and LOC100576174. Target gene expression is measured relative to *mRPL44* and *Rpn2*, which are stably expressed in honeybee ovaries [54]. Gene expression was measured in five biological replicates each consisting of ovaries from multiple individuals: queen (*n* = 3), queen-right worker (*n* = 20), and queen-less workers (n = 10). Differences in gene expression were assessed using a general linear model ANOVA with a Tukey post hoc test and 95% confidence interval. Samples that do not share letters are statistically significantly different with a *p* value < 0.05. All of the sHSP-C genes are significantly different in expression between queen-right worker and queen-less worker ovaries (with the exception of LOC724274) whereas the genes Gmap and LOC100576174 which are immediately adjacent to the sHSP-C are not significantly different in expression between queen-right worker and queen-less worker ovaries. This data validates what we see in the ovary activation RNA-seq dataset comparing sHSP-C gene expression in queen-right worker and queen-less worker ovaries

To gain more resolution of the co-ordinate regulation of this sHSP-C gene cluster in honeybees we performed RT-qPCR and confirmed that the genes of the sHSP-C are differentially regulated in the honeybee ovary in response to QMP (with the exception of *LOC724274*). However, the expression of the adjacent genes that are not related at the sequence-level to sHSP genes; *Gmap* and *LOC100576174* are not significantly different between queen-right ovary and queen-less ovary defining the borders of the co-regulated gene cluster (Fig. 4A). We then assessed how this co-regulation might be occurring by determining if there were binding motifs for Br-C and heat shock elements that are known to regulate the expression of these genes in *Drosophila* [19]. We found that the sHSP-C genes had predicted binding motifs for Br-C and HSE in their promoters, consistent with *Drosophila* [23, 40–42]. To assess epigenetic regulation we examined the sequence for CTCF binding sites and assessed H3K27me3 enrichment (hallmarks of topologically associated domains) at the cluster in queen-less worker ovary, queen-right worker ovary and queen ovary. We found that although the overall levels of H3K27me3 at the sHSP-C are not different between queen-less worker ovary, queen-right worker ovary and queen ovary (Fig. 4B, C), the boundaries of the sHSP-C including the two adjacent non-sHSP genes (*Gmap* and *LOC100576174*) are demarcated by higher levels of H3K27me3 at the 5ʹ and 3ʹ flanks of the cluster which coincides with the predicted CTCF sites. This implies that the sHSP-C cluster in honeybees is co-ordinately regulated by transcription factor binding to promoter sites but also permissive chromatin structure bounded by the CTCF insulator sites, consistent with the sHSP-C acting as a topologically associated domain in the honeybee ovary.

## Discussion

Tracking the evolution of the sHSP cluster in insect genomes is challenging due to the complex phylogenetic relationships of the many sHSP genes in insects. While the presence of a cluster of sHSP genes is widely conserved across insect genomes, phylogenetic evidence indicates that the genes that make up those clusters have arisen in a species independent way, as they are not phylogenetically related across insect orders.

In our analyses we only find support for ancient stable clusters of sHSP genes composed of genes with clear orthologous relationships in Hymenoptera. We find that the majority of genes of the hymenoptera sHSP-C are order-specific and orthologs are not detected outside of the Hymenoptera. Order-specific sHSP-C genes have also recently been identified in a study of eight species of Lepidoptera [29]. Consistent with previous analyses [11] gene conversion cannot explain this phylogenetic signal that we observe in Hymenoptera. Instead our analyses support birth-and-death evolution of this multigene family. We show evidence for sHSP gene loss from the cluster in sawflies, wasps, ants and bees, and we see evidence for duplication in the *Ooceraea* and *Microplitus*. We propose that multiple duplication and loss events during the evolution of the Hymenopteran sHSP-C has led to the complex phylogenetic signal, which depicts multiple lineage specific sHSP-C. Without the ancestral species and/or more closely related non-hymenopteran insect species intermediary to the more basal branching hemimetabolous insects it is not possible to identify the genes that have been lost or duplicated in the insect lineage. It is possible that the evolutionary mechanisms driving the sHSP-C evolution in hemiptera are very different to that of the holometabolous insects, and we cannot rule out that either gene conversion or recent species-specific duplications of the genes in a heavily reduced sHSP-C could be responsible for the phylogenetic signal we see in the hemiptera. However, with the addition of more hymenopteran species, we are able to define the ancestral hymenopteran sHSP cluster and we are able to identify the birth and death events that have occurred in this lineage.

**Fig. 5:**
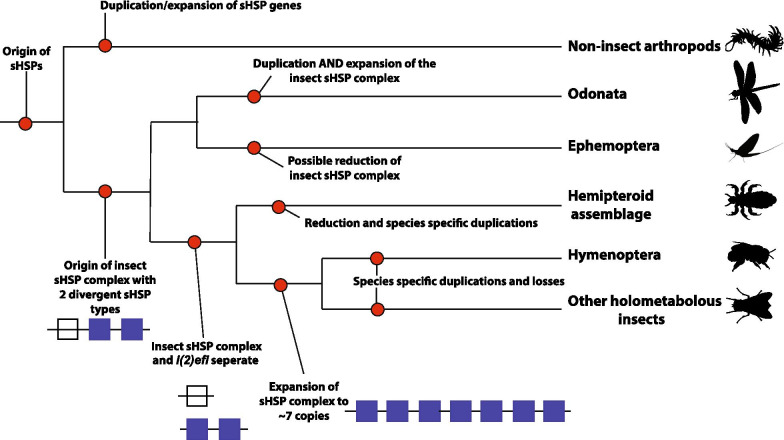
The origin of the insect sHSP-C and *l(2)efl* genes. The origin of the sHSP genes was before the divergence of the Arthropod lineage. The ancestral Arthropod sHSP genes were contained in a cluster. The ancestral insect sHSP-C likely contained two divergent sHSP genes. In the basal branching insect species *Ladona* and *Ephemera* this cluster is retained where there is at least one copy of each of the ancestral sHSP genes (*l(2)efl* and sHSP paralogs). In the higher branching hemiptera the two ancestral sHSPs are separated (*l(2)efl* and sHSP paralogs) and remain so in the holometabolous insects. This separation gives rise to the homologous non-linked insect sHSP *l(2)efl*, which is shared with all insects. After the split of the holometabola from the hemimetabola, the sHSP paralogs undergo extensive duplication and loss events to give rise to the lineage specific sHSP-C genes via birth-and-death evolution

Based on our analyses we propose a model of the ancestral insect sHSP-C which likely contained two divergent sHSP genes (Fig. 5). In the basal branching insect species *Ladona* and *Ephemera* this cluster is retained. In *Ephemera* a sHSP paralog is duplicated to give rise to a sHSP-C containing three genes. In contrast, the *Ladona* genome appears to have undergone multiple duplications of the ancestral insect cluster producing three separate sHSP-C. Each of these clusters contains at least one gene copy of each of the ancestral sHSP genes (*l(2)efl* and sHSP paralog). The existence of the two divergent copies of sHSP in *Ladona* and *Ephemera* is different to that of the rest of the insects’ sHSP-C. It becomes evident in the higher branching hemiptera that these two ancestral sHSPs are separated and remain so in the holometabolous insects. This separation gives rise to the homologous non-linked insect sHSP *l(2)efl*, which is shared with all insects. Evidence of *l(2)efl* sHSP and sHSP-C separation might be seen in the hymenoptera where in some hymenopteran species (*Apis* and *Atta*) the homologous *l(2)efl* gene is retained on the same chromosome as the sHSP-C genes. The genes of sHSP-C present in the hemipteroid insects and holometabolous are paralogous in nature and, after the split of the holometabola from the hemimetabola, it is these sHSP paralogs which undergo extensive duplication and loss events to give rise to the lineage specific sHSP-C genes via birth-and-death evolution.

There is evidence for other gene clusters in insects undergoing birth-and-death evolution. The odorant binding protein family is a multigene family contained within clusters in the genome. In *Drosophila* species these clusters contain a large number of genes ranging between 40 and 61 genes. Phylogenetic analysis and assessment of genome organization in 12 *Drosophila* species showed that this cluster in *Drosophila* is prone to duplication, loss and pseudogenisation, and these specific events could be tracked throughout the 12 species (43).

Our phylogenetic evidence implies that the sHSPs also have a tendency to proliferate, become pseudogenes and vanish. This is supported by the large expansion and multiple sHSP-C that are seen in *Ladona* and the contrasting absence of sHSP-C genes in some hemipteroid genomes. In addition, the existence of the numerous species-specific individual sHSPs that are most likely species-specific duplication events, and the evidence of loss and pseudogenisation in the hymenoptera (*Athalia, Atta* and *Bombu*s), further supports the theory of birth and death evolution as the main mechanism behind the insect sHSP-C evolution. Finally, we do see evidence of recombination of non-sHSP members of the HSP family into the sHSP-C in insects. In Bombyx *HspB1* is an intervening gene in the sHSP-C and in *Frankliniella* *Hsp68* is found immediately adjacent to the genes of the sHSP-C. These data suggest that during the evolution of insects sHSP-C has recombined with other similar regions of the genome and therefore we can not definitively exclude gene conversion.

Interestingly, our phylogenetic evidence also suggests that genes of the sHSP-C remain linked during the evolution of arthropods and insects. In general, arthropod genomes are subject to rapid changes in genome structure in comparison to vertebrates [44, 45] and therefore gene clusters in the arthropods are less likely to remain linked over large amounts of evolutionary time unless there is a selective advantage to do so. For example the E(spl)-C [7, 8] evolved through the co-option of genes to genome location that are co-ordinately regulated and remain linked as a result of functional constraint. However, the sHSP-C genes differ dramatically from E(spl)-C, in that the sHSP-C consists of genes that are paralogous in nature and therefore the evolution and functionality of the sHSP-C is starkly different. The retention of multiple sHSP genes in the genomes of arthropods implies that copy number of the sHSPs is functionally important. Functionally sHsps assemble into large oligomers of 12–32 subunits [46]. Thus copy number in the genome might be functionally constrained to achieve efficient production of these large oligomers. Additionally the genes of the sHSP-C do share regulatory sequences [11] and evidence from expression analyses in *Drosophila* [24], *Bombyx* and in this study in the honeybee support the fact that under certain environmental circumstances the sHSP-C genes are co-ordinately expressed. Thus it is possible that the sHSP-C genes are maintained due to functional constraint. Indeed, Duncan et al. 2020 show that gene clusters that are differentially expressed in honeybee ovaries in response to the loss of QMP are marked by H3K27me3 and that H3K27me3 prefigures the gene expression changes that occur as the bee responds to this environmental stimulus. Although H3K27me3 enrichment at the sHSP-C does not change between honeybee ovary types, we do see demarcation of the sHSP-C by H3K27me3 at the 5ʹ and 3ʹ flank of the sHSP-C which coincides with predicted CTCF sites. Previous studies have shown that H3K27me3 enrichment is found in broad genomic bands marking regions of silent genes and that expressed genes are found preferentially immediately adjacent to the flanks of H3K27me3 enrichment. Although diminished H3K27me3 (a hallmark of open-chromatin) at the sHSP-C alone is not enough to explain the coordinated regulation of the sHSP-C genes during ovary activation, it does suggest that genomic features consistent with coordinate regulation exist around the cluster. Taken together, the results of this study suggest that the sHSP-C genes are maintained in clusters and undergo birth and death evolution, which is driven perhaps by species or order specific selection pressures.

What is clear from this study is that the sHSP cluster is fluid and less conserved in structure that other insect gene clusters. However, its presence in so many insect genomes, and its conserved coordinate expression and possibly regulation, implies that it is an integral genomic component of environmental response in arthropods.

## Methods

### Identification of insect sHSP genes

We retrieved insect, chelicerate, myriapod, crustacean, mollusca sHSP sequences from NCBI, vectorbase (https://www.vectorbase.org/) or the i5K Workspace at the USDA (https://i5k.nal.usda.gov) after identifying them with reciprocal BLASTp [47] searches using Honeybee *l(2)efl* LOC412197. The E-value cutoff for evaluating all sequences in homology search was 10^–10^.

### Phylogenetic analysis

The amino acid sequences of sHSP genes of arthropods (Additional file 1: Table S1) were aligned using clustal X v.1.8 [48]. Using the entire aligned amino acid sequences we reconstructed the phylogenetic relationship of the arthropod sHSP genes using Bayesian methods implemented in MrBayes v.3.1.2. The WAG model [49] of amino acid replacement was used exclusively after testing with mixed models. MCMC chains were run for at least 3,000,000 generations until average standard deviation of split frequencies became stable below 0.05. The first 25% of resulting trees discarded as burnin. The resulting phylogenetic trees were visualised using Figtree [50].

### Motif analyses

CTCF binding sites were located using the Gene Palette program. The sHSP-C and surrounding genes were loaded into the library as sequence, and the consensus sequence for CTCF (CNNNAGNNGGCGC) [51] from *Drosophila* was added as a feature, not allowing for mismatches. Potential binding sites for Br-C and HSE were located using gene sequences from NCBI Gene (http://www.ncbi.nlm.nih.gov/gene) put into Match-1.0 Public (2005), which uses positional weight matrices from TRANSFAC Public 6.0. Settings were altered to minimise false positives, and to use invertebrate sequences. Potential transcription factor binding sites were located in the 500 bp upstream of the transcriptional start site.

### RNA-seq and ChIP-seq analyses

RNA-seq and ChIP-seq data was accessed from Gene Expression Omnibus (GEO), Reference numbers GSE120563 and GSE52291. Fold change for the honeybee sHSP-C genes, homologous *l(2)efl* and non-linked sHSP paralog were extracted from the ovary (GSE120563) and larval (GSE52291) RNA-seq datasets. The heatmap was generated in R studio and was used to visualise the expression values and hierarchical clustering of the honeybee sHSP genes. We used bdgcmp within MACS2 (v2.1.1.20160309) (52) to calculate fold enrichment of ChIP samples relative to input (background) at the sHSP-C in queen-right, queen-less, and queen ovary samples. The grep command was used within the Linux environment to extract the fold enrichment for H3K27me3 from the sHSP-C and the flanking regions. Flanking regions were defined as ½ the length of the cluster as defined by the predicted CTCF binding sites (50 kb to the 5ʹ and 3ʹ of the sHSP cluster). To test whether the levels of H3K27me3 enrichment vary across the sHSP-C, we calculated mean fold enrichment across the 5′ flanking regions, sHSP cluster, and 3′ flanking regions.

### RNA extraction and RT-qPCR

Ovary tissue was removed from queen-right worker (n = 20), queen-less worker (n = 10) and queen abdomens (n = 3) on ice in PBS before snap-freezing on dry ice and storage at − 80 °C. RNA extraction was carried out on biological replicates (n ~ 5) using Trizol reagent (Invitrogen) and purified using RNeasy columns (Qiagen). RNA concentration was determined using the NanoDrop ND1000 spectrometer (NanoDrop). RNA was converted to cDNA using a SuperScript VILO cDNA Synthesis Kit (Invitrogen) as per the manufacturers instructions. For primer design with Primer3 (available online http://frodo.wi.mit.edu [53] gene sequences for the sHSP-C genes, Gmap and LOC100576174 were obtained from NCBI. Primers were checked for specificity using the Primer BLAST program available at http://blast.ncbi.nlm.nih.gov/Blast.cgi. RT-qPCRs were carried out on a BioRad CFX Real-Time PCR detection system with SsoFast EvaGreen PCR mastermix, 5 ng of cDNA, and 300 nM of each primer. The RT-qPCRs were carried out on three biological replicates with each measurement made in duplicate and analysed using the BioRad CFX (CFX Manager software version 3.1). Gene expression was normalized to the geometric mean of two reference genes Rpn2 and mRPL44. Differences in target gene expression were determined by ANOVA (Analysis of variance) with a Tukey’s post hoc test after confirming that the data fit a normal distribution using a Shapiro–Wilk test.

## Supplementary Information

**Additional file 1: Table S1.** Amino acid sequences of sHSP genes of arthropods.

**Additional file 2: Figure S1.** sHSP-C genomic organisation in Arthropods. **Figure S2.** sHSP-C genomic organisation in Hemimetabolous insects. **Figure S3.** sHSP-C genomic organisation in Hymenoptera.

## Data Availability

All sequences and accession numbers (derived from NCBI, vectorbase (https://www.vectorbase.org/) or the Baylor i5K pilot project (https://www.hgsc.bcm.edu/i5k-pilot-project-summary) are available in Additional file 1: Table S1. The dataset(s) supporting the conclusions of this article is(are) available in the Gene Expression Omnibus (GEO) repository, GSE120563 and GSE52291 (https://www.ncbi.nlm.nih.gov/geo/query/acc.cgi?acc=GSE120563 and http://www.ncbi.nlm.nih.gov/geo/query/acc.cgi?acc=GSE52291).
