# Supplementary material for: Evolution and genomic organization of the insect sHSP gene cluster and coordinate regulation in phenotypic plasticity
Source: BMC Ecol Evol. 2021 Aug 4;21:154. doi: 10.1186/s12862-021-01885-8 (PMC8336396; doi:10.1186/s12862-021-01885-8)
Supplement: Supplementary file 2 — Additional file 2: Figure S1. sHSP-C genomic organisation in Arthropods. Figure S2. sHSP-C genomic organisation in Hemimetabolous insects. Figure S3. sHSP-C genomic organisation in Hymenoptera. [file 12862_2021_1885_MOESM2_ESM.docx]

**Evolution and genomic organization of the sHSP gene cluster in Arthropods**

Figures S1-S3

**
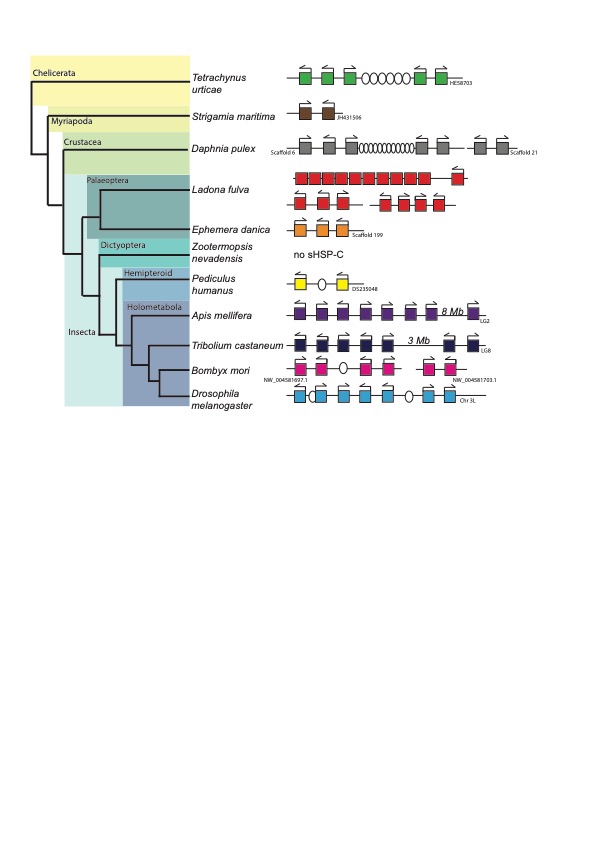
Figure S1. sHSP-C genomic organisation in Arthropods.** BLASTx analysis was carried out to identify the sHSP sequences in the genome of *Biomphalaria glabrata* [mollusca] and the genomes the arthropods (*Tetrachynus urticae* [chelicerate]; *Daphnia pule*x [crustacea]; *Strigamia maritima* [myriapoda]; *Ladona fulvata*, *Ephemera danica*, *Zootermopsis nevadensis*, *Pediculus humanus*, *Apis mellifera*, *Tribolium casteneum, Bombyx mori*, and *Drosophila melanogaster* [insecta]). In *Drosophila*, a sHSP-C of seven genes lies on linkage group 3L. There were also two intervening genes identified, *Hsp67Bb* (*CG4456*), is not a HSP and is instead a member of the Rhodanese Homology Domain superfamily which overlaps the hsp22 open reading frame with transcription in the same direction and a non-coding RNA *CR43481*. In *Bombyx* we identified two sHSP-C on chromosomes 27 and 8 consisting of four and two genes respectively. The cluster of four sHSP genes has an intervening gene HspB1 which is orthologous with all other insect HspB1 genes rather than the sHSPs. In *Tribolium* seven sHSP genes were identified on chromosome LG8. However, *TC005338* and *TC006793* were separated from the other five genes by 3 Mb. In *Apis*, eight sHSPs genes were identified on Chr LG2. *Apis* LOC410857 was separated from the other seven sHSP-C genes by 8 Mb. In each of the holometabolous insect genomes we also identify multiple non-linked sHSP. We identified three sHSP-C in the *Ladona fulvata* (dragonfly) genome. One cluster contained 10 genes, another five genes and the third three genes. In contrast, another deeply branching hemimetabolous insect, *Ephemera* (mayfly) has only a single cluster containing three sHSP genes and no other sHSP genes in the genome. Two clustered sHSPs were identified in the hemipteroid body louse *Pediculus* with an intervening gene, the odorant receptor PhOr6. In addition, while two sHSP genes could be identified in *Zooptermopsis* (termite), these were not clustered. In *Daphnia* we identified two sHSP-C. The first sHSP-C contains two sHSP on scaffold 21, arranged in a head to head arrangement. The second sHSP-C contains five genes on scaffold 6. This complex is interrupted by 12 intervening genes. Similarly, in *Tetrachynus* we identified a five-gene sHSP-C on scaffold HE58703 that is also interrupted by six intervening genes. In contrast we identified only a pair
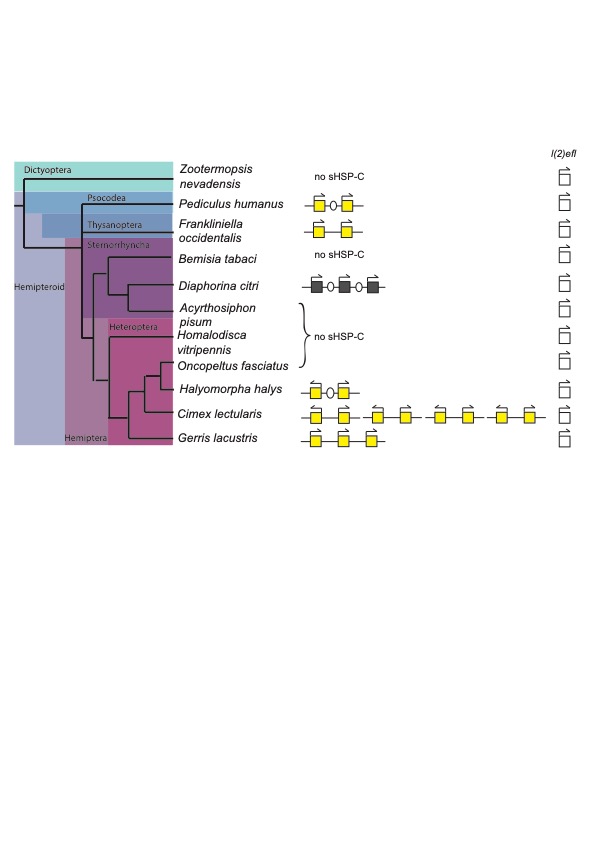
of sHSP in *Strigamia*

**Figure S2. sHSP-C genomic organisation in Hemimetabolous insects.** BLASTx analysis was carried out to identify the sHSP sequences in the genome of *Frankliniella, Acyrthosiphon pisum* (aphid), *Diaphorina, Halyomorpha* (stink bug), *Bemisia* (white-fly), *Oncopeltus* (milk weed bug), *Gerris* (water strider) *. Zooptermopsis* (termite) does not have a sHSP-C. In *Frankliniella* (thrip) a cluster of two sHSP genes was identified, adjacent to these two sHSP was an hsp68 gene homologous to insect Hsp70 family of genes. In a psyllid (*Diaphorina*) two gene clusters were identified with two and three sHSP genes respectively, each with intervening genes. The first cluster of three sHSPs in *Diaphorina* is made up of three tandem repeats of a sHSP and a gene for guanine nucleotide-binding protein subunit beta-5-like. The second cluster in *Diaphorina* consisting of two sHSP genes aligns with the alpha crystallin gene from *Acyrthosiphon pisum* (aphid) and Honeybee and therefore is not considered a sHSP-C (data not shown). In *Cimex* (bed bug) eight-clustered sHSP were found as tandem repeats in a tail-to-tail orientation. Two clustered sHSP genes were also found in *Halyomorpha* (stink bug) separated by an intervening gene. Three sHSP genes were found in a cluster in the *Gerris* (water strider) genome. sHSPs clusters could not be identified in the genomes of Hemipteran insects including *Acyrthosiphon* (pea aphid)*, Bemisia* (white-fly) *Oncopeltus* (milk weed bug) and *Homalodisca* (glassy-winged sharp shooter). Once again, multiple non-linked sHSP paralogs (not shown) were found in the genomes of all hemipteroid insects and all hemipteroid insects had one copy of the homologous insect *l(2)efl* gene.


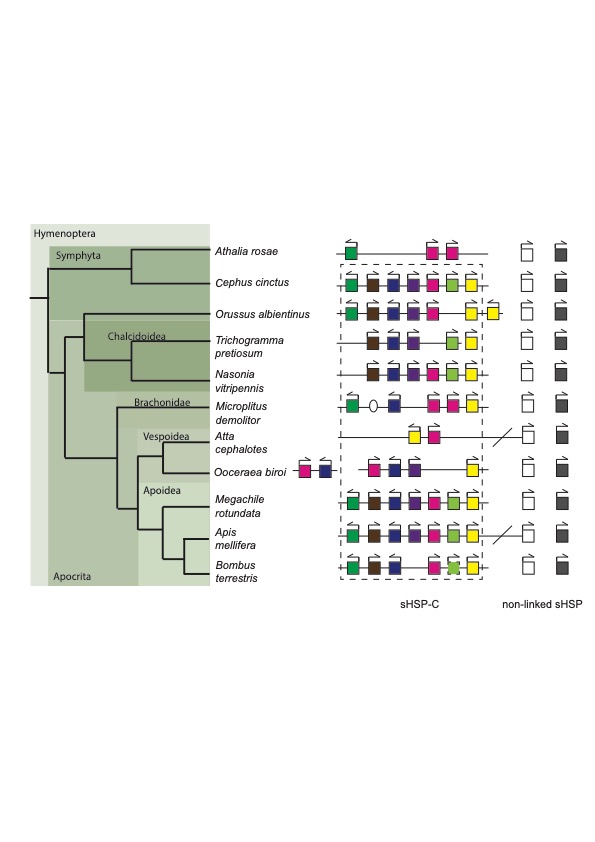


**Figure S3. sHSP-C genomic organisation in Hymenoptera.** BLASTx analysis was carried out to identify the sHSP sequences in the genomes of *Hymenopteran species; Athalia* rosae (turnip sawfly), Cephus cinctus (wheat stem sawfly), *Orussus albientinus* (wood wasp), *Trichogamma pretiosum* (wasp), *Nasonia vitripennis* (parasitoid wasp), Microplitus demolitor (parasitoid wasp), Atta cephalotes (leaf-cutter ant), *Ooceraea biroi* (clonal raider ant), *Megachile rotundata* (leaf-cutting bee), *Apis mellifera* (honeybee), *Bombus terrestris* (buff-tailed bumblebee). We identified five sHSP genes in *Athalia*, three of which were contained in a cluster. In *Cephus cinctus* nine sHSP genes were identified of which seven were found clustered on the genome. Eight sHSP genes were identified in the *Orussus* genome of which seven were linked. Six sHSP genes were identified in the *Trichogamma* genome of which 5 comprised of a cluster. Seven sHSP genes were identified in the *Nasonia* genome of which 6 were linked. Seven sHSP genes were identified in the *Microplitus* genome of which 5 were linked. One intervening gene was also identified separating *XP_008551687.1* and *XP_008551690.1*. Four sHSP genes were identified in the *Atta* genome of which only 2 were linked. Three other ORFs which shared similar sequences to the sHSPs were identified at this genomic location however they were heavily truncated and likely represent pseudogenes and therefore provide evidence for loss of sHSP-C genes in *Atta*. In the ant species *Ooceraea biroi* eight sHSP genes were identified with two sHSP-Cs of four and two genes. The three bee species had a very similar configuration. Nine sHSP genes were identified in the *Apis* genome of which seven were linked. Seven sHSP genes were identified in the *Megachile* genome of which five were linked. Five sHSP genes were identified in the *Bombus* genome of which five were linked. Another ORF was identified within the *Bombus* sHSP-C, however it was characterized as a pseudogene in the NCBI database. In the newest *Bombus* genome release (GCA_000214255.1) the corresponding protein sequence aligns with the respective sHSP-C genes from other hymenoptera.
